# Electromagnetic Wave Absorption Performance of Carbonized Rice Husk Obtained at Various Temperatures

**DOI:** 10.1002/gch2.201900045

**Published:** 2019-08-27

**Authors:** Gan Jet Hong Melvin, Zhipeng Wang, Qing‐Qing Ni

**Affiliations:** ^1^ Material and Mineral Research Unit Faculty of Engineering Universiti Malaysia Sabah Jalan UMS 88400 Kota Kinabalu Sabah Malaysia; ^2^ Institute of Advanced Materials Jiangxi Normal University 99 Ziyang Avenue Nanchang City Jiangxi Province 330022 China; ^3^ Department of Functional Machinery and Mechanics Shinshu University 3‐15‐1 Tokida Ueda 386‐8576 Japan

**Keywords:** carbon, electromagnetic wave absorption, rice husks, silicon carbide

## Abstract

Agricultural wastes such as rice husks (RHs) are valuable due to their feasibility to be converted into carbon materials, low cost, and abundancy in contrast to the conventional carbon material sources. In this study, RHs are carbonized at various temperatures from low to high temperatures, and their electromagnetic (EM) wave absorption properties are evaluated. Carbon materials, silicon carbide (SiC) whiskers, and SiC particles are obtained from RHs carbonized at 1500 °C (CRH1500) for 0.5 h with presence of Ar gas at 1 atm. In order to evaluate their EM wave absorption performance, complex permittivity and permeability are measured by using vector network analyzer, and the values are utilized in the reflection loss (R.L.) calculation according to the transmission line theory. CRH1500, 40 wt% with thickness of 1.6 mm exhibits minimum R.L. of ≈−55.4 dB (>99.9997% absorption) at 11.37 GHz and response bandwidth (R.L. < 10 dB, > 90% absorption) of 4.21 GHz. Low‐cost and abundant RHs, carbonized at various temperatures, show significant absorption performance. Their absorption performance and response bandwidth are highly dependent on matching thickness, indicating that they can be easily modulated for promising electromagnetic wave absorber materials.

The disclosure of unnecessary or excessive electromagnetic (EM) wave emission are expected due to the extensive utilization of devices/applications associated with EM wave especially at GHz frequency ranges (G‐, X‐, Ku‐band, etc.) in electronic fields, wireless communication, and radar.^1^, ^2^, ^3^, ^4^, ^5^ Furthermore, this might leads to device malfunction, disturbance of electronic system, harm the environment, or affect the health of human beings, which has attracted the urgency to develop EM wave absorbing materials.^1^, ^2^, ^3^, ^4^, ^5^ Effective EM wave absorber materials will be able to dissipate EM wave energy into heat or different forms of energy and in result the EM wave cannot be reflected or permeated through the absorber materials, in addition to other advantageous properties such as lightweight, tunable absorbing ranges, and multifunctionality.^1^, ^5^ The incident wave through the absorber materials undergoes the following phenomenon: penetration, reflection, and absorption, for the energy loss process.^1^

Recently, alternative functional absorber materials derived from biomass materials or agro‐based waste materials such as rice husk,^4^, ^5^ walnut shell,^6^ cotton,^7^ spinach,^8^ loofah^9^ have received considerable attention. To the best of our knowledge, the EM wave absorption performance of rice husk (RH) carbonized at high temperatures which produced heterogeneous materials including carbon materials and ceramic materials (silicon carbide whiskers and particles) has not been reported before. RH is an agro‐based lignocellulosic waste material that is abundant in various countries, mainly generated from rice milling activities.^10^, ^11^, ^12^ In year 2016, based on the report from Food and Agriculture Organization of the United Nations (FAO), world rice production is ≈741 million tons. RH is accountable for around 20% from the rice production, which is ready to be utilized.^13^ Commonly, RH is abandoned, manipulated as low value energy resource, or basically combusted at the site, which hinder its effective utilization and unfavorable to the environment.^14^

RHs are lignocellulosic materials, which is beneficial to convert them into raw carbon materials together with silicon content through carbonization process using heat treatment.^15^, ^16^ For instance, Fang et al. reported that carbonized RHs combined with magnetic cobalt particles showed reflection loss of −40.1 dB with thickness of 1.8 mm.^5^ Moreover, Su et al. extracted silicon carbide (SiC) particles from the RHs and they also showed considerable EM wave absorption.^17^ In this study, low cost and abundantly available RHs are simply carbonized at various temperatures, which produced a mixture of carbon and ceramic materials. Accordingly, their complex permittivity and permeability were measured and utilized in reflection loss calculation in order to evaluate the EM wave absorption performance. The combination of heterogenous materials, carbon and SiC, can incorporate the properties of the components to form effective EM wave absorber materials.

The dried RH samples went through carbonization at various temperatures of 800, 1500, and 2200 °C. Typically, the carbonization of raw RHs was at 800 °C, 1 h by utilizing furnace, in argon gas atmosphere. Next, the RHs carbonized at 800 °C were once more loaded into graphite box; further heat treated at 1500 °C, 0.5 h by utilizing graphite resistance furnace operating with the supply of argon gas at 1 atm. Similar process was repeated for the carbonization of RHs at 2200 °C. Finally, the carbonized RHs were crushed into powder within few micrometer range, and they will be denoted as CRH, 800 °C carbonized RHs will be denoted as CRH800, 1500 °C carbonized RHs will be denoted as CRH1500, and 2200 °C carbonized RHs will be denoted as CRH2200, hereafter.

Their structures and morphologies were observed using field emission scanning electron microscope (FE‐SEM; Hitachi SU‐8000) and transmission electron microscope (TEM; JEOL JEM‐2100F) with an accelerating voltage of 15 and 200 kV, respectively. Energy dispersive X‐ray spectroscopy (EDS) equipped together with TEM was utilized to check the elemental compositions. Samples for the complex permittivity and permeability measurement were fabricated by incorporating the CRH in paraffin wax with a weight fraction of 40 wt%. Then, the powder–wax composites were compressed into a 1.0 mm thickness of toroidal shape using a mold designed with an outer diameter of 7.0 mm and inner diameter of 3.0 mm. The complex permittivity and permeability were measured by utilizing a vector network analyzer (37247D, Anritsu Co. Ltd.) in the frequency range of 0.5–13.9 GHz. The reflection loss was calculated by using the measured complex permittivity and permeability.

FE‐SEM image of the CRH1500 is depicted in **Figure** **1**a. A mixture of various shapes graphitized carbon materials, SiC whiskers, and particles were observed. EDS results of CRH1500 further confirmed the existence of carbon and silicon elements, as presented in Figure 1b. The EDS mapping is provided in Figure S1 (Supporting Information). The copper (Cu) peaks are attributed to the copper grid utilized during TEM observation. The inset of Figure 1b shows the magnified FE‐SEM image of CRH1500 carbon porous structure. TEM image of CRH1500 graphitized carbon layers with planar distance, *d* = 0.35 nm, which is slightly larger than (002) plane of single crystal graphite (*d* = 0.335 nm), is shown in Figure 1c. Larger *d* indicates the graphitized carbon is turbostratic carbon.^18^ TEM images of CRH1500 SiC whiskers are presented in Figure 1d,e, and CRH1500 SiC particles in Figure 1f. The diameter of SiC whiskers is up to ≈100 nm with few µm lengths, and the diameter of SiC particle agglomerates is few hundred nm with irregular shapes. From Figure 1e, *d* = 0.25 nm is corresponds to SiC.^17^ From our previous study and other reports, CRH800 are amorphous carbon with porous structure, and the contents included silica (SiO_2_).^10^, ^19^ Meanwhile, CRH2200 contain graphitized carbon materials and mostly SiC particles.^19^ TEM image of CRH800 and FE‐SEM image of CRH2200 are provided in Figure S2 (Supporting Information). Overall, CRH1500 showed most heterogeneity when compared to other CRHs. The process to achieve the heterogeneity is relatively simple, without any further steps to combine different types of materials, in order to obtain excellent EM wave absorption.

**Figure 1:**
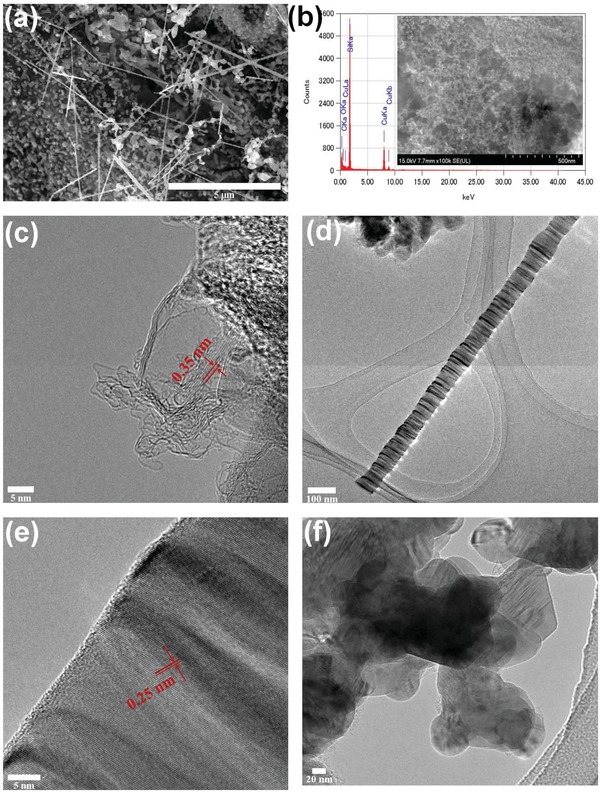
a) FE‐SEM image of CRH1500, b) EDS of CRH1500 (inset: FE‐SEM of porous carbon), TEM images of c) CRH1500 graphitized carbon, d,e) CRH1500 SiC whiskers, and f) CRH1500 SiC particles.

The complex permittivity (real: ε′, imaginary: ε″) and complex permeability (real: *µ*′, imaginary: *µ*″) of the CRHs, in the range of 0.5–13.9 GHz are presented in **Figure** **2**a,b, respectively. As the frequency increased, the complex permittivity declined. This can be attributed to the dipoles in CRHs are not maintained in the phase orientation with the electric vector of the penetrating EM field.^3^ Comparatively, CRH1500 exhibited highest complex permittivity in contrast to other CRHs. Heterogeneous materials including porous carbon, SiC whiskers, and particles exist in CRH1500, which permit strong polarization to take place. Furthermore, SiC might facilitated dielectric loss attributable to the semiconductivity.^20^ Based on the free electron theory, higher ε″ values are proportional to higher conductivity values.^20^ Thus, relatively higher electrical conductivity of CRH1500 permits strong polarization to take place, dissipation of electrostatic charges, Ohmic losses, or multiple scattering attributed to the heterogeneity and large specific area, which lead to improved complex permittivity.^3^, ^21^ It is worth noticing that certain conductive materials are suitable for electromagnetic interference (EMI) shielding material, where the core process of EMI shielding is to produce a blockage made of electrically conductive materials that attenuates radiated or conducted EM energy through reflections and absorption.^22^ Moreover, multifunctional electrical conductive carbon‐based composite materials in various forms, also exhibited competitive performances, such as enhanced electrical conductivity and excellent EMI shielding capability.^23^, ^24^ For the complex permeability, fluctuations can be traced throughout the measured frequency range. This might be attributed to scattered magnetic field due to porous structure (similar with electric field, associated in EM wave propagation), leads to partial energy attenuation.^8^ For CRH1500 and CRH2200, the complex permeability slightly increased until a certain frequency, after which it started to decline. This behavior can be related to the natural resonance or eddy currents loss initiated from the graphitized carbon.^3^, ^25^, ^26^ Graphitized carbon in an alternating magnetic field will build up a close induced current inside the sample, which would scatter the energy and known as the eddy current loss.^25^ Considerable high electrical conductivity of the CRH1500 and CRH2200 might leads to permeability declining swiftly at high frequency, which might be attributed to the eddy current loss.^3^ Furthermore, the SiC which possess semiconductivity that might contributed to a certain resistivity, playing a part that also directs to the decrease of eddy currents when stimulated by the EM waves.^3^, ^26^

**Figure 2:**
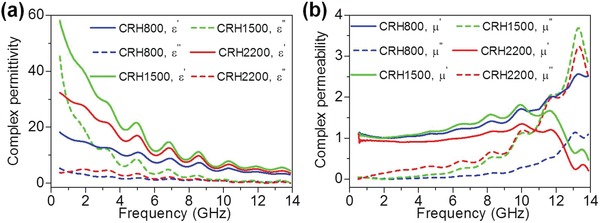
a) Complex permittivity and b) complex permeability of the CRHs.

Based on the transmission line theory, the EM wave absorption performance was evaluated by calculating the reflection loss (R.L.) with the consideration of measured complex permittivity and permeability as follows

(1) $$R.L.\;=\;20\log\left|\frac{Z_{\mathrm{in}}-1}{Z_{\mathrm{in}}+1}\right|$$

where the normalized input impedance (*Z*_in_) is given by the formula

(2) $$Z_{\mathrm{in}}\;=\;\sqrt{\frac{\mu_{r}}{\varepsilon_{r}}}\tan\;h\left[j\left(\frac{2\pi fd}{c}\right)\sqrt{\mu_{r}\varepsilon_{r}}\right]$$

where ε_r_ = ε′ – *jε*″, *µ*_r_ = *µ*′ – *jµ*″, *f* is the EM wave frequency (Hz), *d* is the thickness of the absorber (m), and *c* is the velocity of light in free space (m s^−1^). The R.L. of CRH800, CRH1500, and CRH2200 with weight fraction of 40 wt% and thickness, *t* = 1.0 mm is illustrated in **Figure** **3**a. Apparently, the CRH1500 showed the best EM wave absorption performance when compared with other CRH samples with similar thickness and weight fraction. The CRH1500 indicated minimum R.L. of ≈−17.8 dB (>98.34% absorption) at 12.90 GHz, with response bandwidth (R.L. of <−10 dB, >90% absorption) of 2.52 GHz (11.38–13.90 GHz). Comparatively, CRH800 which consists of amorphous carbon and SiO_2_ showed low R.L. of ≈−5.6 dB. Furthermore, CRH2200 which consists of graphitized carbon and mostly SiC particles showed slightly lower EM wave absorption performance and response bandwidth, with minimum R.L. of ≈−16.3 dB at 13.54 GHz and response bandwidth of 2.35 GHz. Notably, CRH heterogeneity which varied with carbonization temperatures are highly associated with the EM wave absorption performance.

**Figure 3:**
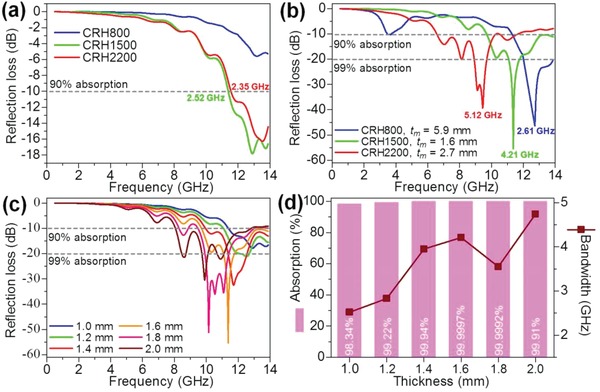
a,b) Reflection loss of CRHs, c) reflection loss of CRH1500 with various thicknesses, and d) absorption performance and response bandwidth of CRH1500 with various thicknesses.

The CRH1500 are thinkable as a heterogeneous material, where graphitized carbon, SiC whiskers, and particles will stimulate additional dielectric relaxation by supplementary dielectric interfaces and higher polarization charges at the interface between those materials.^3^ Moreover, the considerable EM wave absorption performance is also assisted by space charge and orientational polarization. The heterogeneity presents at the interface between the graphitized carbon, SiC whiskers, and particles is strongly correlated to the space charge polarization; meanwhile the orientational polarization is linked with the bound charges (dipoles) present in the CRH1500.^3^ Furthermore, the heterogeneity of CRH1500 will boost the connectivity between the materials and increase the complexity of EM wave propagation paths through the CRH1500. These enable them to polarize repeatedly at high frequency EM wave field, which results in the EM wave energy transformed into other form of energy such as heat energy.^3^, ^5^ In the same time, appropriate complex permittivity and permeability are also important to improve EM wave absorption, which in this study CRH1500 exhibited highest complex permittivity compared to the other CRH samples.^3^, ^21^, ^25^ This also can be associated to another significant parameter related to R.L., i.e., the concept of matched impedance. This concept implies that the intrinsic impedance of the material should be almost identical to that of the free space to attain zero reflection on the front surface of the absorber.^3^ The porous structure of CRH1500 could enhance the impedance matching, where more EM wave can penetrate into the absorber, which will promote the multi reflections that are related to the decay of EM wave energy.^27^ Undoubtedly, to produce optimal absorber with a high minimum R.L., suitable matched impedance is essential.

The absorber's thickness is also a key factor that can influence the R.L. by relating to Equation (2), thus we explored the association between the thickness and the R.L. The R.L. of CRH800, CRH1500, and CRH2200 with matching thickness is depicted in Figure 3b. The R.L. of −10 and −20 dB is matched to 90% and 99% absorption, respectively.^3^ Obviously, CRH1500 with matching thickness, *t*_m_ = 1.6 mm demonstrated highest minimum R.L. of ≈−55.4 dB (>99.9997% absorption) at 11.37 GHz, with response bandwidth of 4.21 GHz. CRH1500 can be considered as the best EM wave absorber compared to other CRH samples, although CRH2200 showed wider response bandwidth of 5.12 GHz, considering CRH2200 *t*_m_ = 2.7 mm which is almost double the *t*_m_ of CRH1500. Furthermore, the dependence of CRH1500 R.L. with various thicknesses is shown in Figure 3c,d. With increasing thickness, the R.L. peak shifted to lower frequency. Noticeably, the minimum R.L. decreased gradually when the thickness increased beyond 1.6 mm. However, wider response bandwidth can be obtained; for instance, CRH1500 with thickness of 2.0 mm showed response bandwidth of 4.74 GHz and in the same time covered almost the whole X‐band. Partially, when the thickness of CRH1500 is beyond 1.2 mm, some parts showed absorption <−20 dB or >99% absorption. Through this particular thickness design of CRH1500, they showed significant EM wave absorption performance and considerable wide absorption frequency. The CRH samples are a prominent EM wave absorber, due to their absorption and response bandwidth that can be exploited simply by adjusting the thickness to suit practical utilization in diverse frequency ranges.

In brief, heterogeneous materials including graphitized carbon, SiC whiskers, and particles were obtained through carbonization of RHs at 1500 °C. Their heterogeneity results in high interfacial polarization, which enhanced the EM wave absorption performance. CRH1500, 40 wt% with thickness of 1.6 mm revealed minimum R.L. of ≈−55.4 dB (>99.9997% absorption) at 11.37 GHz and response bandwidth of 4.21 GHz. Evidently, numerous factors such as type of filler, thickness, and heterogeneity, play a significant role towards the considerable EM wave absorption performance and response bandwidth.

## Conflict of Interest

The authors declare no conflict of interest.

## Supporting information

Supplementary
