# Supplementary material for: Electromagnetic Wave Absorption Performance of Carbonized Rice Husk Obtained at Various Temperatures
Source: Glob Chall. 2019 Aug 27;3(11):1900045. doi: 10.1002/gch2.201900045 (PMC6827532; doi:10.1002/gch2.201900045)
Supplement: Supplementary file 1 — Supplementary [file GCH2-3-1900045-s001.pdf]

Copyright WILEY-VCH Verlag GmbH & Co. KGaA, 69469 Weinheim, Germany, 2019.

Global Challenges

Open Access

Supporting Information

for *Global Challenges*, DOI: 10.1002/gch2.201900045

Electromagnetic Wave Absorption Performance of Carbonized Rice Husk Obtained at Various Temperatures

Gan Jet Hong Melvin, Zhipeng Wang, and Qing-Qing Ni*

Copyright WILEY-VCH Verlag GmbH & Co. KGaA, 69469 Weinheim, Germany, 2019.

Supporting Information

Electromagnetic Wave Absorption Performance of Carbonized Rice Husk Obtained at Various Temperatures

Gan Jet Hong Melvin, Zhipeng Wang, Qing-Qing Ni*

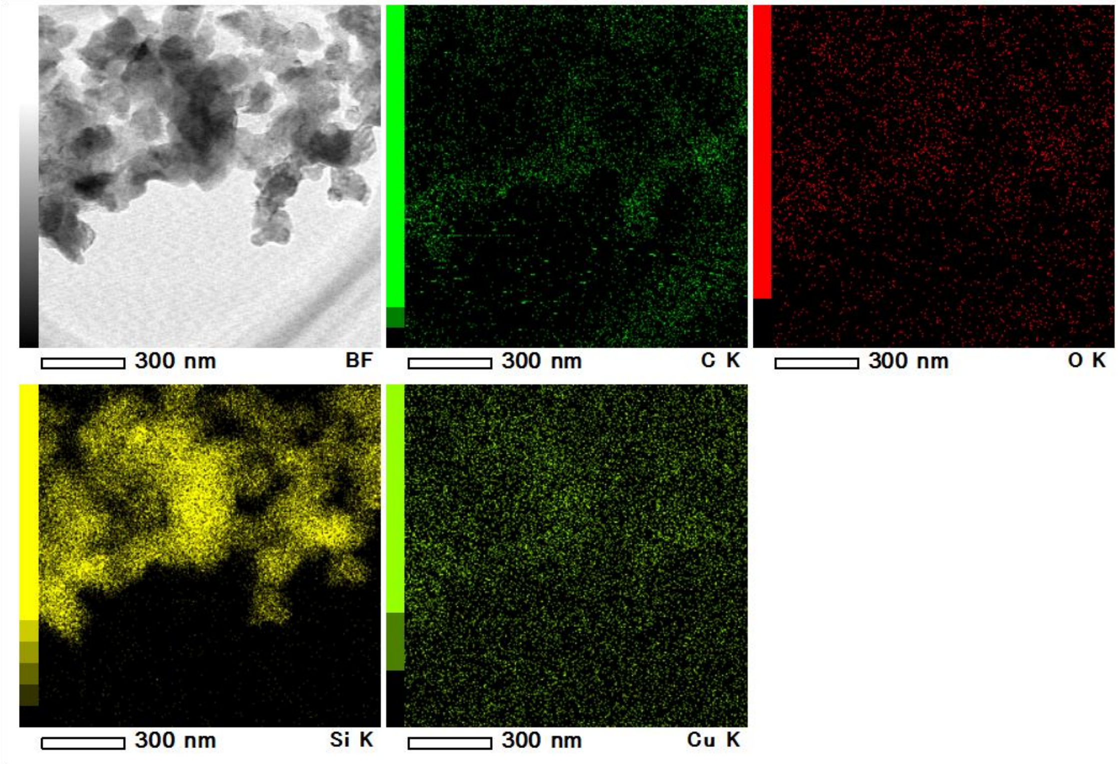

Figure S1. EDS mapping.

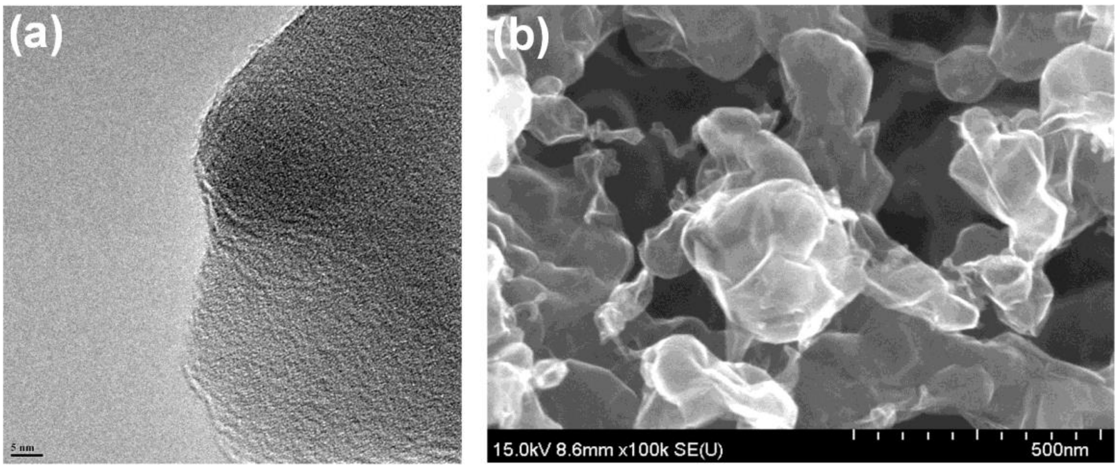

Figure S2. (a) TEM image of amorphous CRH800 and (b) FE-SEM image of mostly SiC particles in CRH2200.
